# Improving the last mile delivery of vaccines through an informed push model: Experiences, opportunities and costs based on an implementation study in a rural district in Uganda

**DOI:** 10.1371/journal.pgph.0002647

**Published:** 2024-10-28

**Authors:** Pamela Bakkabulindi, Solomon T. Wafula, Anthony Ssebagereka, Rogers Sekibira, Aloysius Mutebi, Jimmy Ameny, Christabel Abewe, John Bosco Isunju

**Affiliations:** 1 School of Public Health, Makerere University College of Health Sciences, Kampala, Uganda; 2 Health Support Initiatives, Kampala, Uganda; 3 Landsat ICT Solutions, Kampala, Uganda; 4 Ministry of Health, Kampala, Uganda; 5 Health Economics Division, School of Public Health & Family Medicine, University of Cape Town, Cape Town, South Africa; University of Cape Town, SOUTH AFRICA

## Abstract

Developing countries face challenges in ensuring equitable, timely, and efficient vaccine availability at health facilities. In Uganda, the distribution of vaccines from district stores to the last-mile health facilities is hindered by an unpredictable and unreliable mixed push-pull delivery system. This system often results in poor vaccine management, stock-outs, and missed vaccination opportunities. This pilot study aimed to enhance the efficiency of last-mile vaccine delivery by implementing an informed push model. The specific goals were to improve vaccine lead time, standardize cold chain management during transportation, and evaluate the costs of implementing the informed push model. A mixed methods approach was used to evaluate the impact of the informed push model in Gomba district, Uganda. Both quantitative and qualitative data were collected at baseline and endline. Quantitative data included mode, frequency, lead-time, and costs of vaccine delivery, vaccine stock status, and cold chain maintenance during transportation, gathered through semi-structured interviews. Qualitative data on experiences and challenges were collected using a guide. Descriptive statistics were used for quantitative data analysis, while an ingredients approach was used for costing data. Thematic analysis was applied to qualitative data. The informed push system significantly improved vaccine delivery efficiency and quality in Gomba district. The average lead-time for vaccine delivery reduced from 14 days at baseline to 5 days at endline. Timely vaccine receipt at health facilities increased from 36.8% to 100%. Temperature monitoring during transit improved from 26.3% to 100%. The proportion of facilities experiencing stock-outs dropped from 79.0% to 36.8%. Monthly distribution costs decreased from $494.8 ($0.07 per child) to $445.9 ($0.06 per child). The informed push model is a cost-effective strategy for improving last-mile vaccine delivery by reducing lead times, enhancing cold chain management, and decreasing stock-outs. Integration into the national immunization program is recommended for broader adoption in Uganda.

## Background

Immunization is one of the most cost-effective public health interventions, saving at least two to three million lives each year [1–3]. Sub-Saharan Africa (SSA) faces a disproportionately higher burden of vaccine-preventable diseases, which cause over 2.4 million deaths among children annually due to limited access to life-saving vaccines [4].

Many vaccines can now be obtained at low costs and in mass quantities thanks to cutting-edge technology built on many years of research and development and the help of international organizations [2, 5]. Over the last decade, in developing countries, there have been heightened efforts from the United Nations International Children’s Emergency Fund (UNICEF), Global Alliance for Vaccines and Immunization (GAVI), and local governments that have increased vaccine purchases to protect the population from vaccine-preventable diseases [6].

Unlike common medicinal drugs, vaccines are highly sensitive to temperature changes and thus need to be maintained at appropriate temperature ranges, outside of which they lose potency [7].

Globally, the Expanded Programs on Immunization (EPI) rely on well-functioning immunization supply chain systems to ensure that potent vaccines reach the end user in an equitable, timely, and efficient manner [8]. An efficient immunization supply chain remains a critical element in ensuring access to primary health-care services [9]. The ultimate goal and measure of any successful immunization supply chain system is the increased visibility and availability of potent vaccines at the last-mile health facility level. This goal remains hard to attain for many developing countries [10].

Two factors are critical to a functional end-to-end immunization supply chain and logistics systems: (i) a reliable transport system, and (ii) the maintenance of cold chain status of vaccines during storage and transportation [11]. Firstly, a reliable transportation system ensures that vaccines reach the end user on time. Lessons can be learned from multi-national companies such as Coca-Cola and Johnson & Johnson that have demonstrated their dependability of a reliable transport system for the delivery of goods to the last user [12]. Secondly, a well-maintained cold chain system remains one of the chief cornerstones of a successful vaccination program. This is essential not only for achieving high vaccination coverage (the proportion of individuals receiving a vaccine) but also for ensuring the overall effectiveness and safety of the immunization process. Without a good cold chain system, high vaccination coverage is null and without effect [13]. From production to use, vaccines need to be maintained within a certain temperature range [7]. The recommended temperature range is +2°C to + 8°C for refrigerator vaccines, -50°C to—15°C for freezer vaccines, and -90°C to—60°C for ultra-low temperature vaccines [11].

Like many SSA countries, Uganda struggles to attain the required immunization coverage due to inefficient supply chain systems that often lead to vaccine shortages, missed opportunities for vaccination, and resultant vaccine-preventable disease outbreaks [6, 14]. Indeed, disease outbreaks are largely due to gaps in herd immunity, which are a consequence of the under-vaccination of the susceptible population [6]. Under-vaccination, defined as the suboptimal receipt of vaccination as per the recommended schedule, often results from inefficient vaccine supply chain systems. This can lead to vaccines failing to reach the intended recipients, shortages, or damage due to improper temperature control during transit stockout [6]. As new vaccines are developed and made available to immunization programs, there is a dire and critical need to reduce vaccine wastage, and improve efficiencies, reliability, and agility in the immunization supply chain system [15]. Experiences from the recent COVID-19 pandemic have demonstrated a critical need for resilient immunization supply chain systems.

The Ugandan EPI is largely a vertical program that is situated within the Ministry of Health [16] and has the mandate to provide safe, potent, and effective vaccines for all children and women of childbearing age to reduce morbidity, mortality, and disability due to vaccine-preventable diseases [17]. Since 2012, the key functions of vaccine forecasting, and procurement have become shared responsibilities between the EPI and National Medical Stores (NMS). However, other roles, including storage at the national level and distribution to all district vaccine stores countrywide, were given to NMS to capitalize on their larger storage and transportation infrastructure. At the district level, the district cold chain technicians (DCCT) assume responsibility for cold chain equipment maintenance as their primary role, though they also support proper vaccine storage and handling and, in some cases distribution of vaccines directly to the health facilities.

**Fig 1:**
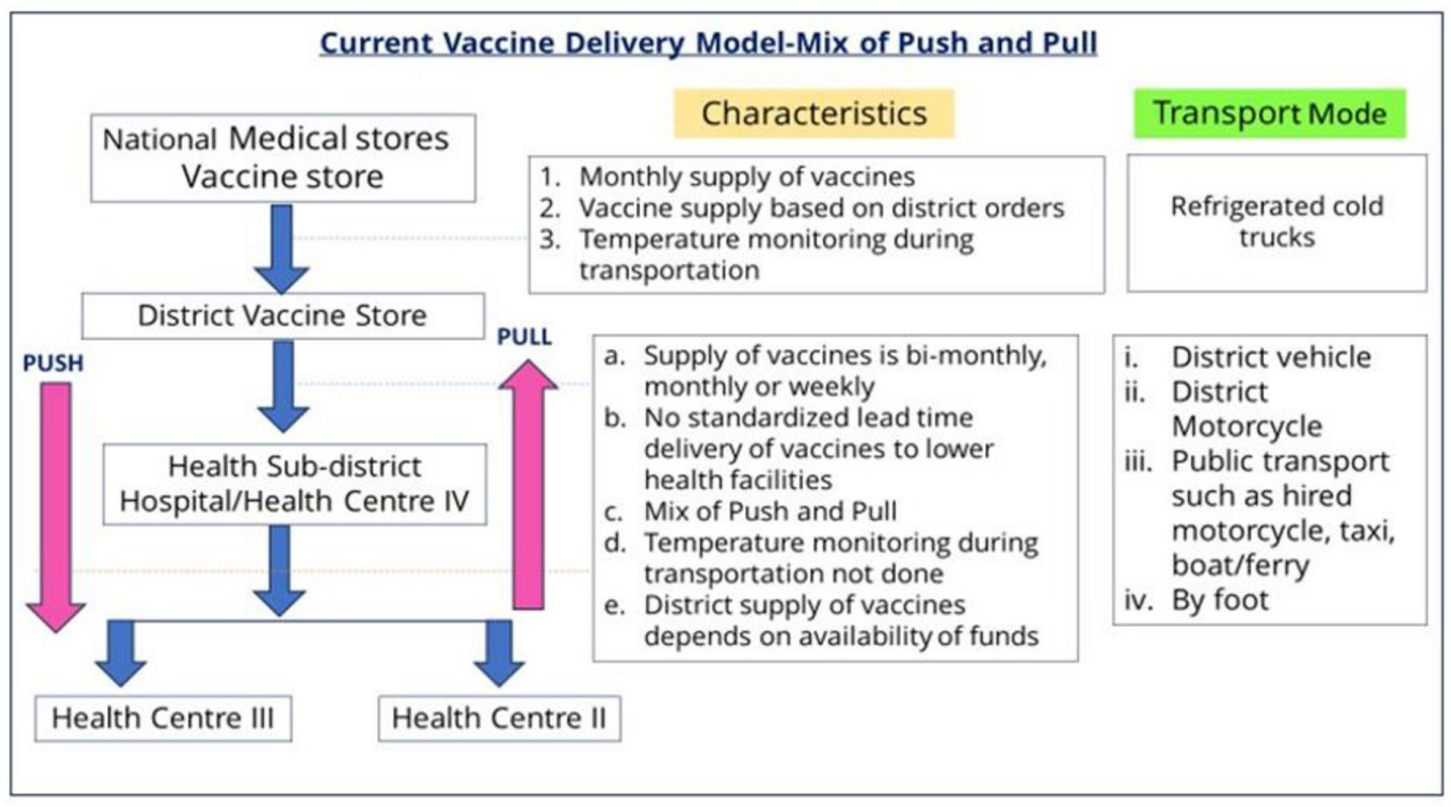
Current vaccine delivery model.

Although Uganda has made considerable improvements in its vaccine supply chain, there is still room to improve its vaccine delivery at the last mile (from the district vaccine store to the health facility level). Several bottlenecks are reported to prevent the reliable distribution of vaccines and immunization supplies at the last mile [16]. In the current Uganda vaccine delivery model (Fig 1), the National Medical Stores executes the delivery of vaccines up to the district vaccine store (DVS). From the DVS to health facilities, there is a mix of push and pull system, with the pull system (health facilities picking their stock) being the dominant one (at 85%) [16]. This ad hoc collection system often results in inconsistent availability of vaccines at the DVS, which may result in shortfalls at other health facilities [16].

The currently practiced mixed system of push and pull vaccine delivery is unpredictable, unreliable, and often presents issues of poor vaccine management, vaccine stockouts and missed opportunities for vaccination at health facilities. The district has no visible role in the equitable distribution of vaccines to each health facility. Therefore, the district is unable to accurately forecast and quantify its vaccine needs and only depends on the information from the health facility forecasts. Moreover, with this model, the supervision of vaccine management by the DCCT at the health facilities is hardly done [16]. A key financing gap cited at the district level is the lack of funds to transport vaccines from the district vaccine store to health facilities [17].

There is a paucity of information on the intricacies, opportunities, and costs involved in the last mile delivery of vaccines in Uganda. There is a need to establish the number of days required to distribute vaccines, the experiences of cold chain maintenance during the transportation of vaccines, and the costs involved in the last mile vaccine delivery of vaccines. The overall aim of the pilot study was to assess the effect of implementing an informed push model of vaccine delivery on improving the efficiency of the last-mile delivery of vaccines. Specifically, the study aimed to assess the effect of improving vaccine lead time -the time interval between the day the vaccines are ordered and the day they are collected at the health center; the standardization of cold chain management practices during vaccine transportation; and the evaluation of the costs associated with implementing this delivery model.

## Methods

### Study setting

The study was implemented in a rural district called Gomba, located in the central region of Uganda, 95 km from the capital district, Kampala. The study was conducted between February and August 2022. As of 2020, Gomba district had a population of 169,518 people; 34,243 children under-five years; 19 health facilities offering vaccination services; Measles Containing Vaccine (MCV) coverage of 55%; Bacillus Calmette-Guerin (BCG) vaccine coverage of 65%; and had experienced 2 measles outbreaks in that same year.

Uganda’s health care system has four levels: hospitals and health centers II, III, and IV. Hospitals are higher-tier facilities with consultant physicians and specialized services. Health centers II, III, and IV are lower-tier facilities (or primary health care) that provide basic services at parish, sub-county, and county levels, respectively. For instance, health center IIs offer outpatient consultations, health center IIIs offer some inpatient care and some laboratory services, and health center IVs offer cesarean deliveries and blood transfusion services [18]. However, all these facilities offer immunization services.

### Study design

This was a pilot intervention study that consisted of before and after comparison studies. It employed both quantitative and qualitative methods of data collection. After the collection of data at baseline, we implemented an informed push model of vaccine delivery in all 19 government-owned health facilities offering immunization services in the Gomba district.

### Description of the informed push model intervention

**Fig 2:**
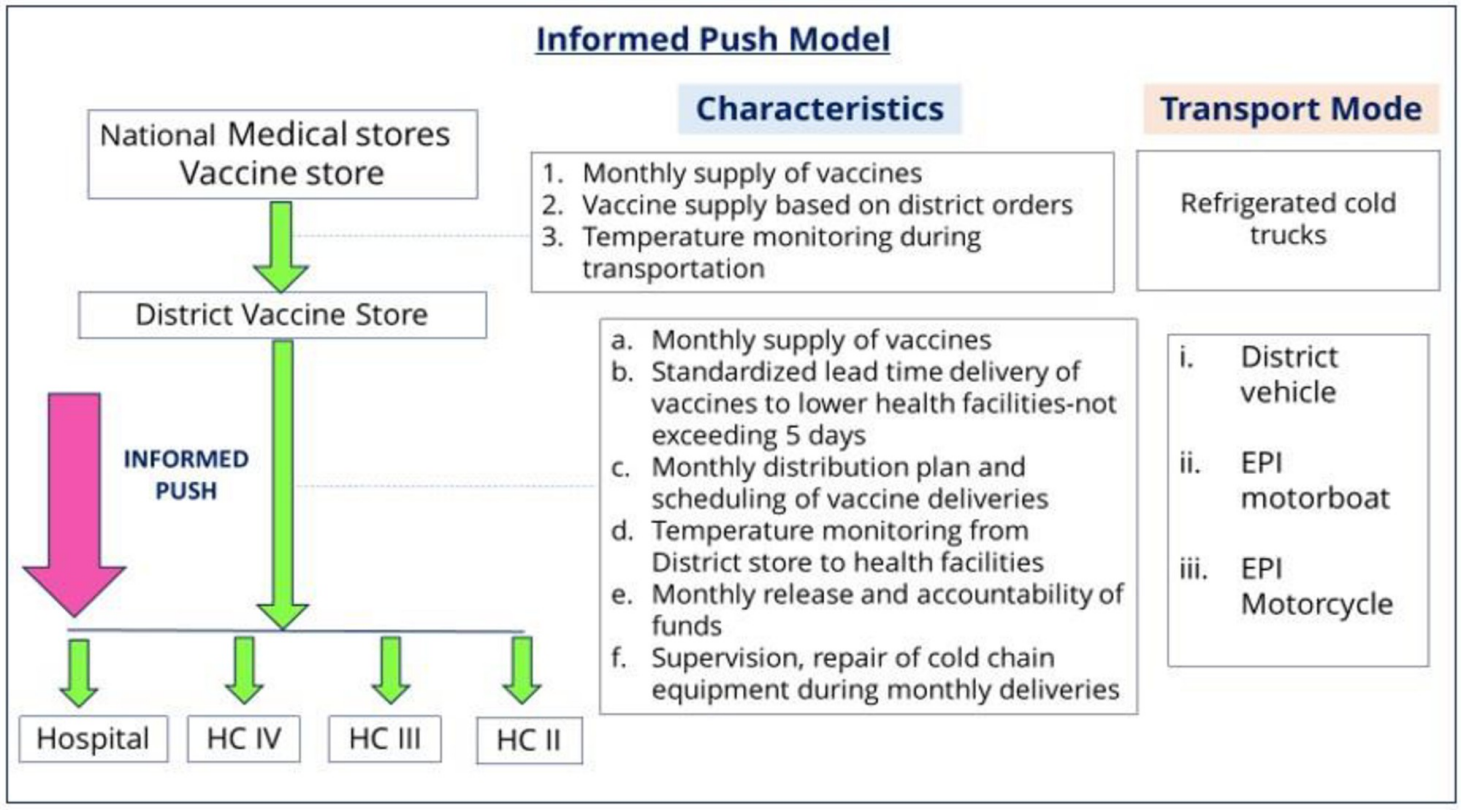
Informed push model.

The informed push model (IPM) intervention was implemented in the 19 health facilities providing immunization services in the Gomba district from March to May 2022. With this intervention, the vaccines were delivered monthly from the District Vaccine Store (DVS) to all 19 immunizing health facilities (Fig 2).

The IPM model differs from an ordinary push model in that it includes the element of first obtaining information on vaccine stock status from the EPI health worker before delivery of the vaccines. During the implementation of the IPM, the DCCT conducted the following tasks: 1) contacting all 19 health facilities to determine and confirm the status of their vaccine stock; 2) designing a distribution plan and strategy for vaccine delivery and thereafter moving according to this planned schedule to each health facility; 3) maintaining the cold chain of vaccines through the use of conditioned ice packs; 4) monitor and record the temperature of vaccines at specified intervals (at the start of the journey and the point of delivery at a health facility). In this study, during the transportation of vaccines, the acceptable temperature range was considered +2°C to +8°C and any temperatures outside this range had to be flagged to the study investigators; 5) recording of the routes used, fuel consumed, and kilometers traveled; 6) documenting the lead-time delivery of vaccines, which is described as the number of days taken to deliver vaccines to all health facilities in each month.

For the intervention period, funds were given monthly for a period of 3 months to cover costs for 1) fuel for transportation of vaccines; 2) safari day allowances for the vaccine delivery team of 3 people who were DCCT, District cold chain assistant (DCCA), and driver; 3) car maintenance costs; and 4) airtime for communication and coordination with the health facility teams. There was no need for vehicle hire as there was an existing district-owned vehicle tagged for immunization activities.

### Data collection

Experienced research assistants were trained for 5 days to administer the quantitative survey questionnaire and qualitative guide. Quantitative and qualitative data was collected before (February 2022) and after (August 2022) the implementation of the study interventions. The study targeted a total of 20 respondents, including one DCCT based at the district level and 19 EPI health workers based at the health facility level, for both the survey and qualitative interviews.

Quantitative survey data was collected using a pre-tested structured questionnaire. Questions included sociodemographic characteristics; vaccine management training; mode of vaccine delivery; vaccine delivery schedules; lead-time delivery of vaccines; vaccine ordering; vaccine stockouts; functional cold chain equipment; cold chain management during vaccine transportation; and costs involved in vaccine delivery and pick-up.

The qualitative guide included questions on the processes and experiences of; vaccine delivery and pick-up; management of cold chain during vaccine delivery and pick-up; vaccine stockouts; costs involved in vaccine pick-up and delivery; challenges and opportunities for last mile vaccine delivery.

### Outcomes measures

The primary outcomes of the study were the lead-time delivery of vaccines, the costs incurred to deliver vaccines to every health facility, and the maintenance of vaccine cold chain temperature.

The secondary outcomes included the frequency of vaccine stockouts, the functionality of health facility cold chain equipment, the preferred mode of vaccine delivery, and the number of health workers mentored on vaccine forecasting and management.

### Data management and analysis

#### Quantitative data

Quantitative data was collected using the Kobo toolbox (Kobo Inc., Boston, MA, USA) and exported to Excel. Then the data was reviewed for completeness, coded, and later exported to Stata 15 (Stata Crop., College Station, TX, USA) for analysis. Frequencies, means, and standard deviation (SD) were used to summarize the data. The analysis was stratified by pre-intervention (February 2022) and post-intervention (August 2022). Due to the small sample size of respondents (as this is a pilot study), comparisons were based solely on changes in frequencies and not hypothesis tests.

The analysis of the costing data used an incremental ingredients approach [19, 20] where each resource input was identified, quantified, and valued by multiplying the number of inputs by their unit prices. A provider’s perspective was considered, and the cost analysis is presented by two cost centers: (a)the costs incurred by health facilities as they pick vaccines, and (b) the costs incurred by the district vaccine store for delivery of vaccines.

Given that an incremental costing approach was employed, the study did not consider already sunk-in indirect health system costs such as human resources, buildings, the purchase of capital equipment, volunteer time, and other opportunity costs. Therefore, the resource inputs considered for the incremental cost analysis were those additional resources that would be required to facilitate the informed push model for vaccine distribution including transport, fuel, vehicle hire, repairs and maintenance costs, and communication costs.

Cost data was initially captured in Ugandan currency (shillings) and later converted to US Dollars (USD). The exchange rate at the time of the study was 1 USD, equivalent to 3,700 Ugandan shillings as of 2022.

#### Qualitative data

We manually analyzed the qualitative data arising from the key informant interviews using thematic analysis. Audio tape recordings were used to capture the experiences and perspectives of the interviewed health workers. These were then all transcribed verbatim into English by the same research assistants who conducted the interviews while preserving the local language concepts. In keeping with this approach, the transcripts were read and re-read to obtain a nuanced understanding of the data. A thematic framework was used and we started by writing memos in the form of short phrases, ideas, or concepts arising from the data in the margins of the text [21]. These were then organized according to specific categories (themes). This was followed by highlighting and sorting out quotes and making comparisons within and between cases. The quotes were then lifted from their original context and re-arranged under the newly developed themes [21]. Finally, the data was interpreted based on internal consistency, frequency and extensiveness of responses, specificity of responses, and trends or concepts that cut across the various discussions. The analyzed data was then presented in text form.

### Ethical considerations

The study was approved by the Makerere University School of Public Health Higher Degrees, Research, and Ethics Committee (SPH-2021-138: A) and the Uganda National Council of Science and Technology (HS2183ES). Permission to carry out the study was received from Gomba district leaders. Written consent was obtained from each respondent.

## Results

The study intervention was conducted for all nineteen health facilities offering immunization services in Gomba district. The same participants were interviewed before and after the study, with no attrition. As seen in Table 1, the majority of the respondents were EPI focal persons (42.1%) who were enrolled nurses by profession. Most of the respondents (73.7%) had been trained in vaccine management in the past five years. The health facilities visited were mostly of level III (47.4%) and level II (47.4%). Many of the health facilities (26.3%) were within 21–30 kilometers, and notably, two health facilities (10.5%) were within the farthest distance of 71–80 kilometers. Most of the health facilities (89.5%) were government-owned (Table 1).

**Table 1:** Sociodemographic characteristics of respondents in Gomba district, Uganda.

Characteristics	Number of participants N (%) N = 19
***Designation of respondents***	
EPI Focal Person	8 (42.1)
Health Facility In-Charge	6 (31.6)
Others*^1^	5 (26.3)
***Profession of respondents***	
Enrolled Nurse	8 (42.1)
Midwife	4 (21.1)
Nursing Assistant	3 (15.8)
Registered Nurse	2 (10.5)
Other*^2^	2 (10.5)
***Ever received any training on vaccine management practices in the past 5 years***	
Yes	14 (73.7)
No	5 (26.3)
***Years in service provision of health workers*, mean (SD)**	
In general health service	10.74 (6.67)
In immunization service	9.37 (6.94)
***Type of Health Facility***	
Health Centre II	9 (47.4)
Health Centre III	9 (47.4)
Health Centre IV	1 (5.3)
***Health facility ownership***	
Government	17 (89.5)
Private	2 (10.5)
**Distance of the health facilities from the district headquarters**	
0–10 kilometers	1 (5.3)
11–20 kilometers	3 (15.8)
21–30 kilometers	5 (26.3)
31–40 kilometers	4 (21.1)
51–60 kilometers	2 (10.5)
61–70 kilometers	2 (10.5)
71–80 kilometers	2 (10.5)

*^1^Others included Health assistant, enrolled nurse, enrolled midwife, vaccinator, and laboratory assistant

*^2^Others included a Health assistant and laboratory assistant.

The same participants were interviewed before and after the study with no attrition.

### Mode and frequency of vaccine delivery

As seen in Table 2, at baseline, a mix of push and pull modes of vaccine delivery existed. The pull model was the dominant mode of vaccine distribution among health facilities (79%), and the push model was the least used one 7/19 (36.8%). Our intervention was an informed push model implemented in all 19 health facilities.

**Table 2:** Mode and frequency of delivery of vaccines in Gomba district, Uganda.

	N (%)	N (%)
Characteristics	Baseline	Endline
***Mode of delivery of vaccines to the health facilities***		
Health Facility Picks from district	15 (79.0)	0 (0.0)
The district delivers to Health Facility	7 (36.8)	19 (100)
Health Facility picks from another Health Facility	5 (26.3)	0 (0.0)
***The person involved in pick-up of vaccines from District vaccine store***		
Health Facility In-charge	10 (52.63)	0 (0.0)
EPI Focal person	10 (52.63)	0 (0.0)
Other*^1^	5 (26.32)	0 (0.0)
DCCT		19 (100)
**Mode of transport used to pick up vaccines from the district vaccine store**		
Hired Motorcycle	10 (52.6)	0 (0.0)
District Vehicle	5 (26.3)	19 (100)
District motorcycle	1 (5.3)	0 (0.0)
Walking	1 (5.3)	0 (0.0)
Other*^2^	2 (10.5)	0 (0.0)
**Preferred mode of vaccine delivery**		
The district delivers to a health facility	15 (79.0)	18 (94.7)
Health facility picks from the district vaccine store	3 (15.8)	1 (5.3)
Both	1 (5.3)	
**Frequency of delivery of vaccines from the district vaccine store to the Health facility**		
Weekly	2 (10.5)	0 (0.0)
Monthly	12 (63.2)	19 (100)
Every two months	1 (5.3)	0 (0.0)
Other*^3^	4 (21.1)	0 (0.0)

*^1^Other included motorcycle rider; neighbor at the health facility; a health worker from another health facility; any health worker present at the health facility

*^2^Other included a bicycle and use of public transport means (taxi)

*^3^Other included getting vaccines from a health center IV due to lack of a functional fridge; In a quarter we receive vaccines two times; Picks from another facility when there’s a need; Picks from another health facility

At baseline, the health facility in-charges (52.63%) and EPI focal persons (52.63%) were involved in vaccine pick-up. However, we note unqualified persons (26.3%) picking vaccines, such as motorcycle riders. During the intervention, the DCCT was the one delivering vaccines to all 19 immunizing health facilities as per the IPM (Table 2).

The reported common mode of transport used to pick up vaccines from the district vaccine store at baseline was hired motorcycles (52.6%); however, with the IPM intervention, the existing district vehicle earmarked for vaccination activities was used for vaccine distribution to all 19 health facilities (Table 2).

At baseline, only 12 (63.2%) facilities reported receiving vaccines at a monthly frequency, and this increased to all 19 (100%) facilities being supplied monthly with IPM.

The majority of health workers at baseline and endline (79.0% and 94.7%, respectively) preferred the district to deliver vaccines (Table 2).

#### Finances and stock out challenges influence the dynamics involved in delivery dynamics

Qualitative interviews also pointed out some difficulties with modes of vaccine delivery and how this was due to limited finances. This also points to consequences such as understocking or sometimes overstocking, as remarked by the district cold chain technician and one EPI focal person:

*“ Before the implementation of the study, most health facilities picked vaccines from the district vaccine store at their convenience. This was mainly because there were no available financial resources to be used for the delivery of vaccines. When health facilities pick vaccines, there is a tendency for them to take more than they need which leads to overstocking on their part and understocking for the health facilities that pick last.”*

(KII, cold chain staff, Gomba district).

*“The reason why we pick vaccines from the district store is because the district does not deliver them to the facility*, *so instead of staying without vaccines*, *one must spend money on transportation to ensure that vaccines are obtained”*.

(KII-12, health worker, Gomba district).

*Informed push delivery*: *A one-stop solution to various challenges at the health facility level*. Respondents also thought IPM may have solved the challenges of understocking in some facilities and overstocking in other facilities as it was common with the earlier mixed push and pull system.

*“From my experience, it is better for the cold chain technician to distribute vaccines because then it allows for efficient distribution, as I would first quantify and confirm the vaccine needs of a health facility before distribution. I was even able to re-distribute vaccines between facilities which prevented over-and under-stocking. I was also able to identify cold chain challenges and solve those I could there and then at the health facility*.*’*

(KII, cold chain staff, Gomba district).

“*The best mode of vaccine delivery is when the district delivers to the health facility. This is because most of the time it is cost-effective because the distance from the health center to the district is long, which means you have to use much transport and you again have to facilitate the person who is going to pick up the vaccine, so it is less expensive when the district delivers. It would cost less if the district supplies all facilities rather than everyone going there to pick up*”.

(KII-4, health worker, Gomba district).

Health workers complained of the inconveniences that are associated with vaccine pick-up which sometimes result in not being able to attend to patients or clients. One respondent remarked as follows: ‘*’Picking up vaccines is inconveniencing for health workers and patients*. *For example*, *a health facility at the level of health center II has three staff so when one leaves the workload increases for the two who have remained at the facility’’*. (KII-9, health worker, Gomba district).

*“Vaccine pickup is difficult for us. First of all, our health facility is in a remote location, and it is far from where you find a taxi, costs three dollars, and again the transport to here is not readily available and is only in the evening, so you end up walking that distance at night’’*.

(KII-5, health worker, Gomba district).

*“When the district makes a delivery*, *we are sure that the vaccines will be received in good condition*. *We also save on transport and reduce the waiting time for mothers because then the health worker does not have her duties to pick vaccines’’*.

(KII-2, health worker, Gomba).

### Vaccine delivery lead-time

Health workers’ understanding of the required lead-time for vaccine delivery markedly improved, with the average estimation decreasing from 22 days at baseline to a correct knowledge of 5 days by endline (Table 3).

**Table 3:** Lead-time delivery of vaccines in Gomba district, Uganda.

	N (%)	N (%)
Characteristics	Baseline	Endline
**Awareness of vaccine delivery schedules from National medical stores to district vaccine store**		
Yes	2 (10.5)	1 (5.3)
No	17 (89.5)	18 (94.7)
**Knowledge of the recommended days within which vaccines should be received at the health facilities upon delivery to the district vaccine store**		
Yes	3 (15.8)	2 (10.5)
No	16 (84.2)	17 (89.5)
**Timely receipt of vaccines at the health facility**		
On-Time	7 (36.8)	19 (100)
Not on Time	12 (63.2)	0 (0.0)
**The stated number of days in a month recommended for vaccine delivery (lead-time), mean (SD)**	22.00 (32.97)	5.00 (00.00)
**Days taken to deliver vaccines to all health facilities as stated by the district cold chain technician, mean (SD)**	14 (00.00)	5.00 (00.00)

Qualitative findings also corroborated this: *“Before implementation of the study, it would take an entire month for the health facilities to pick vaccines from the district vaccine store as and when they had the time or felt it was convenient”. Given that funds were readily available during the implementation, we took 5 days to distribute vaccines to all health facilities.”* (KII, cold chain staff, Gomba district).

The lead-time delivery of vaccines improved from 14 days at baseline to 5 days at endline. Similarly, timely receipt of vaccines at the health facilities improved from 36.8% at baseline to 100% by endline (Table 3).

As seen in Table 3, by baseline and endline, most of the respondents (89.5% and 94.7%, respectively) were still not aware of the vaccine delivery schedules from the national stores to the district vaccine stores.

This information is corroborated by the qualitative interviews:

*“No, we are not aware of the vaccine delivery times, though sometimes we are informed, but at times we just go there, not knowing the vaccine delivery schedule for national medical stores”*

(KII-1, health worker, Gomba).

*“The challenge is that we are not informed about the delivery of vaccines by national medical stores because if we knew, then we would go there at the right time, but unfortunately, we always go to the district vaccine store when the vaccines are already finished”*.

(KII-7, health worker, Gomba).

### Cold chain and vaccine management

Notably, at baseline, only 5 (26.3%) of respondents reported that they were monitoring the temperature of vaccines during transportation. However, during the implementation of the study, the DCCT was mandated to monitor the temperature twice (at the start and end of the journey) of the vaccines during vaccine transportation. This is reflected in the endline results, as all the respondents (100%) attested to temperature monitoring for vaccines during vaccine distribution (Table 4).

**Table 4:** Cold chain and vaccine management processes in Gomba district, Uganda.

	N (%)	N (%)
Characteristics	Baseline	Endline
**Availability of cold chain storage equipment at health facility**		
Yes	17 (89.5)	19 (100)
No	2 (10.5)	0.0 (0)
**Functionality of the available cold chain equipment**		
Yes	14 (73.7)	19 (100)
No	3 (15.8)	0.0 (0)
**Type of available functional Cold Chain Equipment**		
Fridge	17 (89.5)	19 (100)
Vaccine carriers	8 (42.1)	14 (73.4)
Fridge tag	5 (26.3)	11 (57.9)
Cold boxes	2 (10.5)	4 (21.1)
Freezers	1 (5.3)	4 (21.1)
Other*^1^	1 (5.3)	2 (10.5)
**Type of cold chain equipment used for storage of vaccines during transportation**		
Vaccine Carrier	19 (100)	0 (0.0)
Cold box	0 (0.0)	19 (100)
**Type of ice packs used during vaccine transportation**		
Frozen ice packs	13 (68.4)	0 (0.0)
Conditioned ice packs	6 (31.6)	19 (100)
**Temperature monitoring during vaccine transportation from the district vaccine to health facilities**		
Yes	5 (26.3)	19 (100)
No	14 (73.7)	
**Type of temperature monitoring tool used during vaccine transportation for those that carried out monitoring**		
Thermometer	3 (15.8)	0 (0.0)
Fridge tag	2 (10.5)	19 (100)
**Frequency of monitoring temperature during transportation of vaccines from district vaccine store**		
Not Monitored	11 (57.9)	0 (0.0)
Once	3 (15.8)	0 (0.0)
Twice	2 (10.5)	19 (100)
Do not know	3 (15.8)	0 (0.0)
**Issues with vaccines received**		
Yes	3 (15.8)	2 (10.5)
No	16 (84.2)	17 (89.5)
**Type of issues with vaccines received**		
Vaccines with vaccine vial monitor changes	2 (10.5)	1 (5.3)
Poor bundling	1 (5.2)	1 (5.3)
**Health workers’ knowledge of reading and interpreting VVM changes**		
Yes	19 (100)	19 (100)
**Health workers checking for VVM changes at the point of receipt of vaccines**		
Yes	18 (94.7)	16 (84.2) (16)
No	1 (5.3)	3 (15.8)

*^1^ Other included ice packs

More people indicated they did not monitor the temperature before the implementation of IPM.

*“To be honest, I do not monitor for temperature when I go to pick vaccines from the district vaccine store*.”

(KII-17, health worker, Gomba).

*“I won’t lie to you that we do it*. *We use a taxi or motorcycle to pick up the vaccines from the district vaccine store to the health facility*. *So*, *with that mode of transport*, *you can’t monitor the temperature*.”

(KII-2, health worker, Gomba).

*“To be honest, before the implementation of this study, during the vaccine delivery, I would measure the temperatures but not record them anywhere. But during implementation, I was mandated to record the temperatures before, during, and on arrival at the health facilities; this was a big lesson for me!*”

(KII, cold chain staff, Gomba district).

The availability of cold chain equipment for the storage of vaccines improved from 89.5% at baseline to 100% at endline (Table 4). Similarly, the functionality of this equipment improved from 73.7% at baseline to 100% at endline. This is because the DCCT was able to repair broken-down fridges during the monthly delivery of the vaccines (Table 4).

At baseline, only 31.5% of the respondents reported using conditioned ice packs for vaccine carriage ice pack during vaccine transportation. With the DCCT being able to observe the right practices, this improved, and by endline, all facilities (100%) reported the use of conditioned ice packs during vaccine transportation (Table 4).

The number of respondents reporting cold chain issues with vaccines received decreased from 15.8% (3/19) at baseline to 10.5% (2/19). The commonest issue reported was changes in the vaccine vial monitor (VVM). In addition, all the health workers knew how to read and interpret the vaccine vial monitor (VVM) changes and more than 80% of the respondents checked for VVM changes upon receipt of vaccines at baseline and endline (Table 4).

Key informants highlighted some advantages of the IPM approach. One respondent remarked as follows:

*“The push system or when the district delivers vaccines had so many advantages. We were able to identify faulty fridges and have them repaired in a short time. We were able to distribute vaccines according to their rightful quantifications. We were able to train the health workers in quantification, cold chain maintenance, and record keeping. At some point, we even re-distributed vaccines to ensure a good stock balance. We noticed an improvement in the number of children immunized during the 3 months of implementation.’‘*

(KII, cold chain staff, Gomba district).

When asked about the earlier strategy for vaccine delivery at baseline, respondents highlighted the difficulty in maintaining the cold chain during transit, a situation that can affect the vaccine’s potency. One respondent stated as follows:

*“In my opinion, there is no proper cold chain maintenance when we pick up vaccines from the district store. One can go with ice packs in the vaccine carriers. But by the time one gets to the district store, the ice packs have completely melted with hardly any cold temperature. I am not sure that the cold chain status is maintained as we transport these vaccines”*

(KII-5, health worker, Gomba).

### Vaccine stock status

All the respondents at both baseline and endline reported the availability of the issuing and ordering tool (Table 5). The number of health facilities reporting that they did not receive vaccines as ordered decreased from 57.9% at baseline to 26.3% at endline. Similarly, the number of health facilities experiencing vaccine stockouts also reduced from 79% at baseline to 36.8% at endline following the implementation of IPM.

**Table 5:** Vaccine stock status, Gomba district, Uganda.

	N (%)	N (%)
Characteristics	Baseline	Endline
**Availability of issuing and ordering tools at health facility**		
Yes	19 (100)	19 (100)
**Health worker Knowledge of the use of the issuing and ordering tool**		
Yes	19 (100)	19 (100)
**Health Facility receiving vaccines as ordered**		
Yes	8 (42.1)	14 (73.7)
No	11 (57.9)	5 (26.3)
**Health facility experiencing stock out of vaccines**		
Yes	15 (79.0)	7 (36.8)
No	4 (21.1)	12 (63.2)
**Instances of under-fulfilled orders received by health facility**		
*Yes*	12 (63.2)	6 (31.6)
*No*	7 (36.8)	13 (68.4)

As seen in Table 5, all the respondents at both baseline and endline reported the availability of the issuing and ordering tool at the health facility. However, at baseline, more than half of respondents (57.9%) reported that their health facilities did not receive vaccines as ordered, which improved at the endline as only 26.3% reported not receiving vaccines as ordered.

Similar disparities between number of vaccines ordered and supplied were highlighted qualitatively at baseline, as indicated as follows:

*“We usually do not receive the same quantity of vaccines as ordered. Sometimes I reach the district vaccine store and find that some vaccines are out of stock, and I end up taking fewer quantities than what is needed”*.

(KII-8, health worker, Gomba district).

*“Other times on arrival at the district vaccine store, I find most vaccines are taken, and are finished, so I take what is left.”*

(KII-13, health worker, Gomba district).

### Costs incurred during vaccine distribution at baseline and by endline

#### Summary of cost analysis results

The costs incurred by the health facilities and district to distribute vaccines at baseline were $494.8, $0.07 per child, while the costs incurred by the IPM model were $445.9, $0.06 per child. Overall, IPM appears to be cheaper in terms of overall cost (Table 6).

**Table 6:** Summary of costs for distribution of vaccines, Gomba district, Uganda.

Resource input	Pull/Push model	Informed Push model
Costs incurred	(Baseline costs in USD)	(Endline costs in USD)
**1) By the health facilities**		
• Transport/Fuel	170.8	0
**2) By the District**		
• Transport/Fuel	324	270
• Repairs & maintenance	0	81
• Safari Day Allowance	0	81
• Communication	0	14
**Total**	**494.8**	**445.9**
**Cost per child**	**0.07**	**0.06**

Before the study’s implementation, there were no consistent district-budgeted resources for vaccine delivery. However, the average baseline costs were $ 494.8, and these catered for only fuel costs for the delivery of vaccines. The push model required the district cold chain team to deliver vaccines to all 19 immunizing health facilities using the district car that was allocated to the EPI department. There were three personnel involved in vaccine delivery, including the DCCT, DCCA, and driver, who were given daily allowances. The total monthly costs expended for vaccine delivery were $445.9 (Table 6). When we consider the total number of children under one year in Gomba district (7,290), the $445.9 total cost for vaccine delivery under the IPM model translates into a unit cost of $0.06 to reach each infant with vaccination services.

A total of 5 days were used to deliver vaccines to all health facilities following a pre-defined route. On average, 1200 kilometers were traveled within the 5 days of the month’s delivery, and approximately 200 liters of fuel were consumed. Therefore, 6 liters of fuel were required for each kilometer moved in distance. The costs budgeted and incurred included safari day allowance, fuel, communication, and car maintenance costs.

#### Source of funds for vaccine distribution at baseline

**Fig 3:**
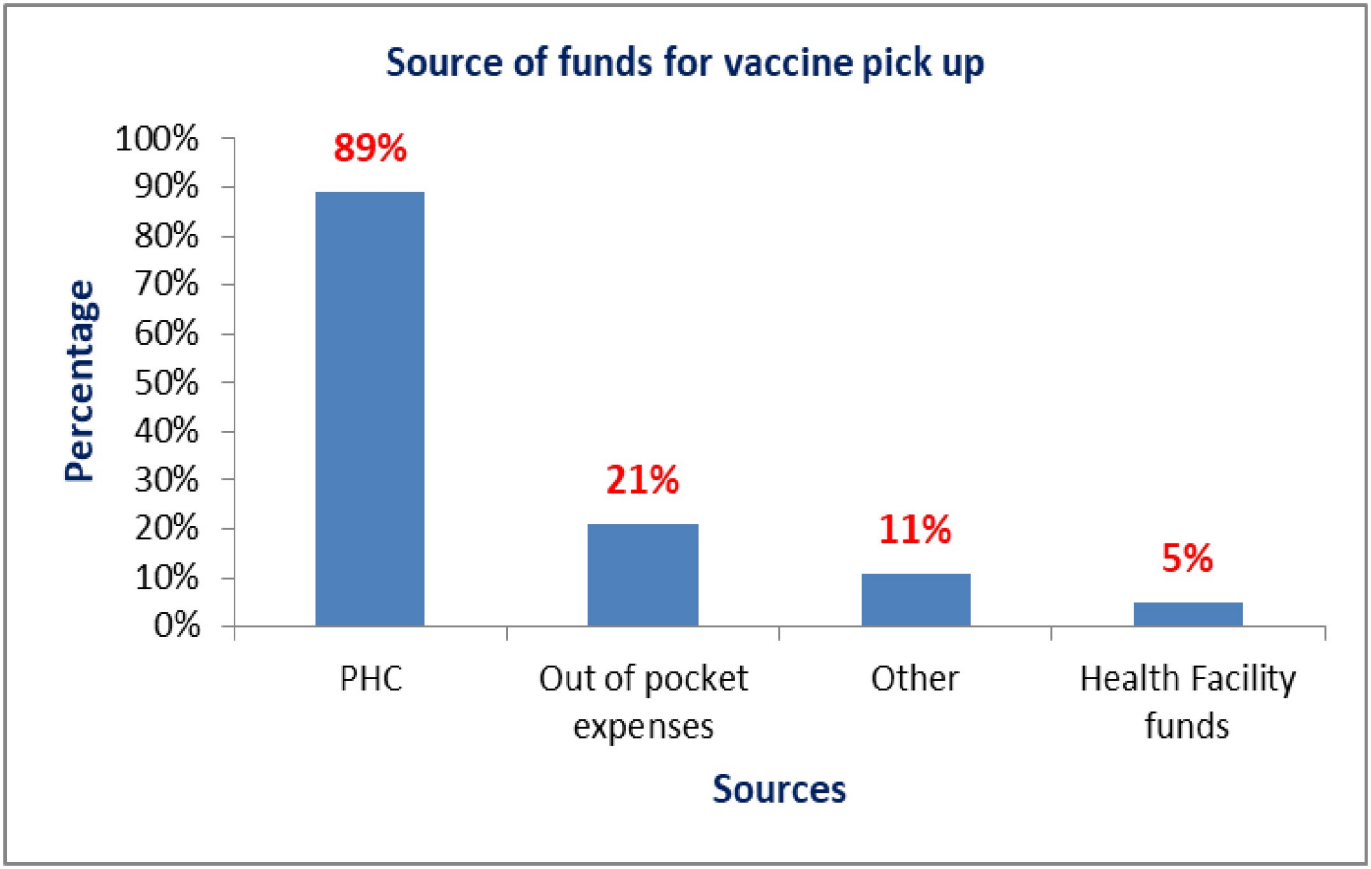
Source of funds for vaccine pick-up at baseline.

At baseline, major areas of expenditure were transport (53%), and fuel (42%). In addition, a significant 32% under ‘other cost’ included cost areas for hiring motorcycles, motorcycle repairs, lunch, and airtime for communication. The respondents indicated that primary health care funds (89%) were the major source of funds for vaccine pick-up. However, 21% of the respondents reported using out-of-pocket funds for vaccine pick-up based on multiple-choice questions asked (Fig 3).

Qualitative interviews also provided perspectives on the source of funds as seen below:

*“The source of funds for transport to pick up vaccines is a charge we put on the clients. We get the money from mothers who come for immunization. Mothers are charged $ 0.3 each time they come for immunization, so it’s the money we use for transport costs during vaccine pick-up”.*

(KII-6, health worker, Gomba district).

As noted, a few financial challenges were highlighted before IPM, as follows:

*“The main challenge with vaccine delivery is lack of funds and consistency in the flow of funding because when you have funds at a given point in time and don’t have in the next then vaccine delivery becomes inconsistent. The main issue is having available funds, once one has the funds then regular vaccine distribution becomes feasible”*

(KII, cold chain staff, Gomba district).

## Discussion

This is the first known pilot implementation study in Uganda that has assessed a cost-informed push vaccine delivery model and its potential benefits in improving the last-mile delivery of vaccines. The results of this pilot study can be used for planning and evidence-based response actions. In our study, the intervention included several components that can improve the efficiency of the last mile delivery system, including timely delivery of vaccines, maintenance of the cold chain, and cost of the last mile logistics system.

Our findings show that the lead-time delivery of vaccines from the DVS to all immunizing health facilities was reduced to 5 days each month, and yet before, health facilities could take on average three weeks to pick up vaccines from the district vaccine store. Evidence from this study suggests that when health facilities picked vaccines, there was no real-time temperature monitoring done during transportation, and therefore, no known visibility of active cold chain maintenance performed, which gravely compromised the potency of the vaccines. However, during the IPM intervention, with the use of a trained cold chain technician, cold chain maintenance was enforced, and therefore, vaccines were delivered in their rightful conditions. In addition, there was a reduction in vaccine stockouts as health facilities received vaccines that they were able to consume after proper quantification, as opposed to before when they got vaccines in an ad hoc manner. This study demonstrated varying costs for vaccine pick-up, which oftentimes were either out-of-pocket expenses incurred by health workers or costs that were eventually charged to caregivers before receipt of immunization services. Moreover, at baseline, the pick-up of vaccines by health workers would impinge on their time for delivering immunization services which would lead to missed opportunities for vaccination. At baseline, the costs incurred by districts and health facilities in vaccine distribution were $494.8 ($0.07 per child) as compared to $445.9 ($0.06 per child) with IPM. This shows that our intervention- IPM, is cheaper and yet has numerous tangible and intangible benefits associated with the delivery of vaccines by the DCCT. These benefits include efficient distribution of vaccines, onsite mentorship, repair of fridges, support supervision, response to cold chain challenges in real-time, and time saved for health workers to attend immunization activities.

### Vaccine delivery model

Our results showed that 79% and 95% of health facility workers preferred the push model at baseline and endline, respectively. This finding confirms that health workers prefer to stay at their respective facilities and execute their mandate of delivering healthcare services at the service delivery point rather than having to routinely travel to collect vaccines from the DVS, also recognizing that the majority of the health facilities within this setting are struggling with understaffing and patient overload challenges [22]. This takes the pressure off the health workers involved in picking vaccines from the DVS. The constraints related to low staffing levels at health facilities are not alien and have been widely documented by several scholars, as affecting access to vaccination services [23].

These results are similar to those from a similar study conducted in Nigeria that indicated that a push vaccine delivery model saved one to six hours of the healthcare workers’ time spent on vaccine logistics (collection and distribution) each week. This enabled healthcare workers to focus on delivering services to mothers and children, resulting in a reduction in clients’ waiting times as well as enhanced job satisfaction [10].

Our study showed that 26% (5/19) of the ’others’ category that picked up vaccines included neighbors and motorcycle riders, who represent untrained, unqualified, and unequipped personnel unfit to handle and manage vaccines during transportation. The most common mode of transport used for transporting vaccines from the DVS to the health facilities was hired motorcycles (for more than half of the facilities). These motorcycles were a more preferred mode of transport because they are flexible, convenient, and easily available means of transport regardless of the weather changes, versatile, and relatively less costly than hiring or fueling a car over the same comparative distance to be traveled. In many of these cases, rural areas are disadvantaged due to poor road networks, especially during rainy seasons [24]. However, there are certainly concerns about safety, maintenance of the cold chain for the vaccines, possible theft of vaccines or diversion to other parties. On the other hand, the vaccines could get destroyed in case the motorcycle got into an accident, which is a common and fatal phenomenon in Uganda.

Vaccine handling and management are best handled by trained and skilled workers who can recognize and respond appropriately to temperature excursions [2]. Studies have shown that poor vaccine handling is often associated with technically incompetent stakeholders [13]. The success of the EPI program is attributed to the use of potent vaccines, which depend on good cold chain status; therefore, vaccine management is of critical importance [11].

### Lead time for vaccine delivery

The majority of respondents were not aware of the vaccine delivery schedule by national vaccine stores at both baseline and endline. Our results showed that only 5% of the health workers knew the NMS vaccine delivery time schedules and only 11% knew the correct recommended number of days within which vaccines should be delivered to health facilities upon their receipt at the DVS. This is consistent with a study in Ghana, which showed that out of the 21 respondents, 6 (29%) had heard of the “vaccine supply period,” of which, 2 (33.3%) knew the purpose of the supply period, and could correctly tell the vaccine supply period for their facility store [25].

This suggests that more effort is still needed to bridge the knowledge gap around microplanning and vaccine delivery schedules. It is also important to recognize that health workers at the facility level need to know the vaccine delivery schedule by national medical stores so that their immunization microplan synchronizes with the vaccine delivery schedules, which can help avoid vaccine stockouts [26]. For example, in 2011, the Ministry of Health-Uganda acknowledged that vaccine stockouts were not due to shortages but rather attributed to persistent distribution problems within the districts [27].

### Cold chain and vaccine management

The cold chain remains a highly vulnerable point for EPI programs in developing countries [28]. The core of the vaccine cold chain is temperature monitoring [2]. Since all vaccines need to be maintained within certain temperature ranges, the monitoring of vaccine temperatures is a must, especially during transportation [2]. All health workers reported reading the VVM upon receipt of vaccines at the health facility. These results are consistent with results from various vaccine assessments that show that health workers are knowledgeable about VVM and their use in the protection of vaccines from heat excursions [29]. However, the VVM only captures heat exposure over time and not freezing conditions [2]. Indeed, studies have shown that cold chain management weaknesses are often observed during the transportation and storage of vaccines [28].

Our study showed that only 6/19 (31.58%) health workers used conditioned ice packs during vaccine pick-up. These results are consistent with a study conducted in Ghana, where only 23% of health workers had heard of the “conditioning” ice packs [25].

The DCCT is not only meant to deliver vaccines to the health facility but also supports the vaccine supply chain system with activities like quality assurance of temperature monitoring, cross-checking for good functionality of the cold chain equipment at a facility, mentorship of health workers in vaccine management, etc. The storage vaccine equipment was functional in most of the facilities and by endline, all facilities had functional cold chain equipment. Therefore, maintenance of the vaccine cold chain across the facilities was appropriately executed, including the use of vaccine carriers for storage and picking up the vaccines from the DVS. It is important to recognize that monitoring of the cold chain performance is often limited by a lack of performance management systems, and is rarely done due to the limited prioritization of cold chain assessments, like temperature monitoring studies and funding [30]. However, the gaps in cold chain management are still evident in the transportation of the vaccines from the DVS to the health facilities, with more than 30% of the respondents at endline stating that their facilities do not use frozen ice packs during the transportation of vaccines from DVS to the health facilities. Such gaps in the cold chain management of vaccines during transportation ought to be effectively addressed and managed to ensure vaccine safety, quality, and integrity. Inadequate availability of cold boxes and frozen packs to maintain the cold chain for vaccines is disadvantageous to the health sector as it attracts a loss of up to 55% of the medicines [31].

### Vaccine stockouts

Results from our study show that the number of health facilities experiencing vaccine stockouts decreased from 79% (15/19) at baseline, to 37% (7/19) by endline. These results are similar to a study conducted in Nigeria that showed a reduction of vaccine stockouts from 43% to 0% after streamlining their last-mile vaccine supply delivery to an informed push model [10].

Vaccine stockouts often lead to missed opportunities for vaccination, decrease the number of children who could have been immunized, and create a negative demand for immunization [14]. Missed opportunities for vaccination for children that result from inequitable and poor supply chain systems negatively impact the vaccination coverage and can expose children to morbidity and mortality from vaccine-preventable diseases which in the long run negatively impacts the economy of a country [14].

### Costs involved in vaccine distribution

Our study revealed a total cost of $494.8 incurred at baseline for vaccine distribution, of which $170.8 was incurred by health facilities and $324 by the district. However, it is important to note that the health facility costs are underestimated as they don’t include opportunity costs such as productivity lost when a health worker leaves their duty station to pick up vaccines from the DVS. Furthermore, it is important to highlight that in a context where resources are highly constrained, most health facilities often struggle to mobilize funds for the transportation of vaccines and primarily rely on grossly insufficient primary health care funds and out-of-pocket charges to patients. Collecting money from patients or caretakers to finance the transportation of vaccines, among others, exposes them to financial ruin as they seek healthcare services as a result of increased out-of-pocket expenditures to meet medical and non-medical costs.

For the IPM, our study estimated the monthly cost for implementation of the push model at $445.9, and this translates to an estimated quarterly and annual cost of $1, 337.7, and $5,350.8, respectively. The estimated monthly cost per child reached with vaccination services in the Gomba district was $0.06. This very low per child cost makes financial sense and is a strong case for the nationwide scale-up of the IPM. Moreover, the IPM is cheaper, more beneficial, and saves loss of work productivity than the mix of push and pull model.

If the IPM is scaled up, which we recommend, a national adaptation of this model will require proper forecasting and budgeting at national and district levels and planning for logistical arrangements and needs for all catchment health facilities.

Some studies have used third-party logistic companies to deliver the vaccines to the last mile often at high costs. A study in Cape Town (South Africa), found that outsourcing vaccine logistics to the private sector reduced delivery and inventory costs, improved adherence to temperature thresholds, reduced delivery delays, improved handling practices, and allowed greater volume flexibility [32].

Arguably, the use of third-party company logistics to deliver vaccines to the last mile may be too expensive and not sustainable. Our research shows that a better and more sustainable approach would be to empower the district cold chain teams to use the available designated cars for the EPI programs to distribute vaccines in a timely and equitable manner while offering support supervision in vaccine management, conducting ad hoc cold chain maintenance of vaccine equipment at the health facilities, and ensuring health facilities have the right stocks of vaccines. The DCCT can provide mentorship to health workers in EPI in stock management, and vaccine forecasting while having visibility on the functionality of cold chain equipment at the health facility level.

A robust last-mile cold chain and supply chain system will require technical personnel who can carry out multiple tasks, such as repairs of cold chain equipment, mentorships, support supervision, and vaccine management, in addition to vaccine distribution.

There are some limitations to consider: Firstly, the indirect costs already borne by the government, such as health personnel, opportunity costs, and other capital costs, have not been included in the cost analysis. Secondly, although the study is informative in terms of the potential to use the IPM approach for vaccine delivery, the findings should be treated with caution because they were limited to one district with 19 facilities and the cost of IPM may differ from district to district as it is affected by distances and terrains, hence not generalizable across the country. The third limitation is that the sample size of this pilot study also limits the conduct of appropriate statistical tests, and hence we cannot strongly generalize our findings to other settings. Lastly, the lack of a control group also requires cautious interpretation of findings due to possible confounding and other biases. Future studies should consider using better designs, such as large cluster randomized trials or quasi-experimental designs, to have a more nuanced estimation of the effect of the intervention. Despite these limitations, the study still provides important preliminary information that can guide the design of future studies.

## Conclusion

To our knowledge, the study described here is the first of its kind that has attempted to demonstrate an improved and cost-effective last-mile immunization supply chain model that uses district-based trained cold chain personnel. To address the challenges of an inefficient and poorly defined last-mile vaccine logistics system, our study implemented and costed an informed push model of vaccine delivery from the district to all immunizing health facilities. This streamlined vaccine supply chain system ensured vaccine quality, timeliness of vaccine delivery, reduced stockouts, reduced the burden of HCWs in vaccine pick-up, ensured timely repairs of broken-down cold chain equipment, provided visibility of the stock status of health facilities, provided a platform for onsite mentorship, and other less tangible spillover benefits. The costs, successes, and areas of opportunity described herein can serve as a guide for other last-mile vaccine systems in Uganda and other countries with similar contexts. The monthly costs associated with the IPM ($445.9 and $0.06 per child) make financial sense and a strong case for the model and this is further reinforced by the various benefits of last-mile delivery. This benefit is critical in hard-to-reach areas that tend to have frequent vaccine stockouts, missed opportunities for vaccination, and generally poor vaccine coverage. The national adaptation of IPM will require proper forecasting at national and district levels to ensure logistical arrangements are appropriate for vaccination in designated catchment areas.

## Supporting information

S1 Dataset (XLSX)
